# Secondary Hemophagocytic Lymphohistiocytosis Due to Typhoid Fever

**DOI:** 10.7759/cureus.42175

**Published:** 2023-07-20

**Authors:** Shekhar Shekhar, Rahul Radhakrishnan, Vidya S Nagar

**Affiliations:** 1 Medical Oncology, Tata Memorial Hospital, Mumbai, IND; 2 General Medicine, Grant Medical College and Sir Jamshedjee Jeejeebhoy (JJ) Group of Hospitals, Mumbai, IND

**Keywords:** typhoid fever, secondary hlh, secondary hemophagocytic lymphohistiocytosis, enteric fever, hemophagocytic lymphohistiocytosis, hlh

## Abstract

Hemophagocytic lymphohistiocytosis (HLH) is a potentially fatal hyper-inflammatory state that is caused by a highly activated but ineffective immune system. It can be primary or secondary to triggers like infections, malignancies, and autoimmune conditions. The authors present the case of a young male with a fever and abdominal pain due to typhoid. He continued to have a high-spiking fever and developed dyspnea, requiring oxygen therapy despite being treated with appropriate antibiotics. Laboratory evaluation revealed cytopenias and deranged liver function tests, and abdominal imaging revealed hepatosplenomegaly. These clinical and laboratory findings raised suspicion of HLH secondary to typhoid fever. Further investigations were suggestive of hyperferritinemia and hypofibrinogenemia, and bone marrow aspirates showed hemophagocytes. The patient was treated with immunosuppression (dexamethasone) and antibiotics and showed remarkable recovery. Hemophagocytic lymphohistiocytosis should be suspected in patients with tropical infections like enteric fever, tuberculosis, malaria, dengue, etc. that worsen despite appropriate treatment, as late diagnosis is associated with greater mortality.

## Introduction

Hemophagocytic lymphohistiocytosis (HLH) is a potentially life-threatening disorder caused by uncontrolled immune activation. Fever, cytopenias, splenomegaly, hepatitis, hyperferritinemia, and hemophagocytosis are the key features of this syndrome. It can be primary or secondary. Primary (or familial) HLH is mainly seen in the paediatric age group and is due to genetic defects in the immune system. Secondary HLH is due to triggers like infection, autoimmunity, and malignancy and can occur in any age group [1].

When it comes to secondary HLH triggered by infections, persistent fever, cytopenias, rising liver enzymes, and unresponsiveness to appropriate antimicrobials are the pointers that should raise an early suspicion. However, since typical features of HLH like leukopenia, thrombocytopenia, splenomegaly, and deranged liver function tests are present in tropical infections and sepsis as well, the diagnosis of HLH secondary to infections is often delayed and even missed [2]. It is thus essential to keep a high index of suspicion for HLH in cases of complicated or deteriorating patients with infections or sepsis, as features of HLH might be falsely attributed to infections themselves (particularly tropical infections like dengue, malaria, enteric fever, and tuberculosis). A late or missed diagnosis of HLH carries a high mortality risk [3].

The HLH 2004 criteria (and the more recent Modified HLH 2009 criteria) can be used to arrive at a diagnosis and start early treatment. In addition to these, the H-score is a validated criteria score to estimate the probability of HLH and consists of graded clinical and laboratory parameters. It has a higher sensitivity (90%) [4].

Treatment of HLH is based on the HLH-1994 and HLH-2004 protocols, which have immunosuppressive regimens consisting of multiple elements like corticosteroids, cyclosporine, etoposide, etc. It should be noted that these protocols were developed for primary HLH in the paediatric age group [5]. The treatment of HLH secondary to infections in adults should be individualised and tailored depending on the clinical condition of the patient. Resolution of HLH secondary to infections with appropriate treatment of the infectious trigger alone (without any immunosuppression) has frequently been seen. Corticosteroids (dexamethasone) with or without etoposide may be used if clinical conditions and organ function deteriorate despite infection-directed treatment. The use of etoposide is associated with secondary infections and secondary malignancy [6,7]. Thus, treatment of HLH secondary to infections consists of infection-directed treatment, and immunosuppression is added based on clinical judgement and organ function [7].

The authors present a case of secondary HLH due to typhoid fever in a young adult male. Hemophagocytic lymphohistiocytosis is a rare but serious complication of typhoid fever, and only a few cases have been reported so far [8].

## Case presentation

A 23-year-old male from Mumbai, India, an economics student staying at the college hostel, came to the outpatient department (OPD) with complaints of fever for 15 days, loose motion for 10 days, abdominal pain for four to five days, and vomiting for the last three days. Fever was described as being high-grade, associated with chills, occurring mostly at night, and relieved with oral acetaminophen and wet sponging. Stools were of semi-solid to watery liquid consistency without blood or mucous. Abdominal pain was a dull ache, localised in the peri-umbilical region, without any specific exacerbating or relieving factors or radiation. He had also been having nausea and vomiting for the last two days. He also complained of malaise and body aches since last week. He had not noticed any rash, swelling, lumps, or recent weight loss. He had been consuming food at his hostel mess for the last month. He denied any travel history or history of similar illnesses in family or friends recently. Neither he nor any of his family members had a history of tuberculosis. He only took acetaminophen for fever and had no significant drug history. He denied any addiction or recreational drug use.

On examination, he was conscious, oriented, and cooperative. He was febrile (100.8°F), normotensive (100/60 mm Hg), had a pulse rate of 96 bpm, regular, low volume, a respiratory rate of 22 breaths per minute, and normal O_2_ saturation. There was no pallor, icterus, clubbing, lymphadenopathy, or oedema. There was no rash on the skin or oral mucosal lesion. The abdomen was moving with respiration equally in all quadrants; it was soft and non-tender. There was Hackett's grade 1 splenomegaly (Table 1).

**Table 1:** Hackett's grading system of palpable splenomegaly Table showing Hackett's grading of splenomegaly based on the inferior extent of palpable spleen [9]. The patient had grade 1 splenomegaly on presentation that increased to grade 2 on day three of admission.

Hackett’s grade	Description of splenomegaly
Grade 0	Normal, impalpable spleen
Grade 1	The spleen is palpable only on deep inspiration
Grade 2	The spleen is palpable on the mid-clavicular line, halfway between the umbilicus and costal margin
Grade 3	The spleen expands towards the umbilicus
Grade 4	The spleen goes past the umbilicus
Grade 5	The spleen expands towards the symphysis pubis

The liver was not palpable. Cognitive functions were normal, with no subtle signs of delirium. Neck rigidity was absent. The rest of the systemic examination was unremarkable.

He was admitted for further evaluation and treatment. The patient presented with complaints of fever for 15 days, loose motions for 10 days, abdominal pain for five days, and vomiting for three days. On examination, he was found to have relative bradycardia and Hackett’s grade 1 splenomegaly. Our main differential diagnoses included enteric fever, acute gastroenteritis, abdominal tuberculosis, pancreatitis, dengue fever, and malaria.

On presentation, the patient was empirically started on ceftriaxone 2 mg intravenously (IV) once daily (OD). An ultrasonogram of the abdomen was suggestive of hepatosplenomegaly with a thickened ileocecal junction and enlarged non-necrotic and a few necrotic lymph nodes in the ileocecal region. Malarial antigen, peripheral smear for the malarial parasite, and dengue NS1 antigen/IgM were negative, ruling out dengue fever and malaria. Serum amylase and lipase (repeated at a 48-hour interval) were within the normal limit, ruling out pancreatitis. The chest X-ray did not show any significant abnormalities. The patient continued to have fever spikes with a haemogram suggestive of deteriorating cytopenias even after 72 hours (Table 2).

**Table 2:** A timeline of laboratory investigations and clinical trends (fever, oxygen requirement) relevant to the case on different days after admission The patient's condition (fever, acute respiratory distress syndrome (ARDS)) and laboratory parameters (cytopenias, hepatitis) were seen to deteriorate despite antibiotic escalation. Hence, there was suspicion of HLH secondary to typhoid fever. Dexamethasone was started on day five, following which there was an improvement in the clinical condition and laboratory parameters. Hb: haemoglobin; TLC: total leukocyte count; PLT: platelet count; AST: aspartate transaminase; ALT: alanine transaminase

Patient parameter	Reference range	Day 1	Day 3	Day 4	Day 5	Day 6	Day 7	Day 8	Day 9
Hb (gm/dL)	12-14.5	12.7	12.3	9.6	9.0	10	10	10	10.2
TLC (/cumm)	4,000-11,000	6,200	1,060	1,400	4,200	5,100	10,800	10,200	11,700
PLT (/cumm)	150,000-450,000	128,000	42,000	29,000	38,000	31,000	161,000	171,000	293,000
Total bilirubin (mg/dL)	<1.0	0.5	1.2	1.5	1.2	1.1	1.1	1.0	0.9
AST (U/L)	10-45	51	49	444	281	238	115	85	78
ALT (U/L)	10-45	54.7	57.9	196	176.6	155.4	228	172	114
Serum creatinine (mg/dL)	0.5-1.2	0.6	0.8	1.0	1.1	1.0	0.9	1.0	0.8
Fever spikes		Present	Present	Present	Present	Present	Afebrile	Afebrile	Afebrile
Oxygen requirement		No	No	No	Yes	Yes	Tapering	No	No

Therefore, on day three of admission, antibiotics were escalated to intravenous meropenem 1 gm three times daily (TDS) as extended infusions while blood, urine, and stool cultures were awaited. On the same day, persistent fever, worsening cytopenias, and elevated liver enzymes prompted us to send for investigations with suspicion of HLH (serum ferritin, fasting triglycerides, serum fibrinogen, as well as a bone marrow aspiration and biopsy to rule out other sinister causes of pancytopenia with persistent fever like leukaemia). Serum leptospira IgM and urine leptospira polymerase chain reaction (PCR) done in view of fever with cytopenias and hepatitis were negative and ruled out leptospirosis. Viral markers (hepatitis B surface antigen (HBsAg), anti-hepatitis C virus (HCV) antibodies, HIV antibodies, hepatitis A virus (HAV) IgM, and hepatitis E virus (HEV) IgM) were negative. Although a colonoscopy and a biopsy from the ileocecal region were suggested to rule out intestinal tuberculosis, the patient was not willing. On examination, he now had Hackett's grade 2 splenomegaly, while the liver was palpable two-finger breaths below the subcostal margin in the midclavicular line.

On day five of admission, the patient started complaining of breathing difficulty with arterial blood gas (ABG) (Table 3), suggesting hypoxemia, for which he was started on O_2_ therapy with nasal prongs (4 L/min).

**Table 3:** Arterial blood gas analysis (ABG) of the patient suggests Type 1 respiratory failure paO_2_: partial pressure oxygen in arterial blood; paCO_2_: partial pressure carbon dioxide in arterial blood; sO_2_: oxygen saturation

ABG parameter	Patient value	Reference range
pH	7.48	7.35 - 7.45
paO_2_	53.4 mmHg	75 - 100 mmHg
paCO_2_	30.9 mmHg	35 - 45 mmHg
Bicarbonate	26.9 mmol/L	22 - 26 mmol/L
sO_2_	91.8%	95 - 100%

**Figure 1:**
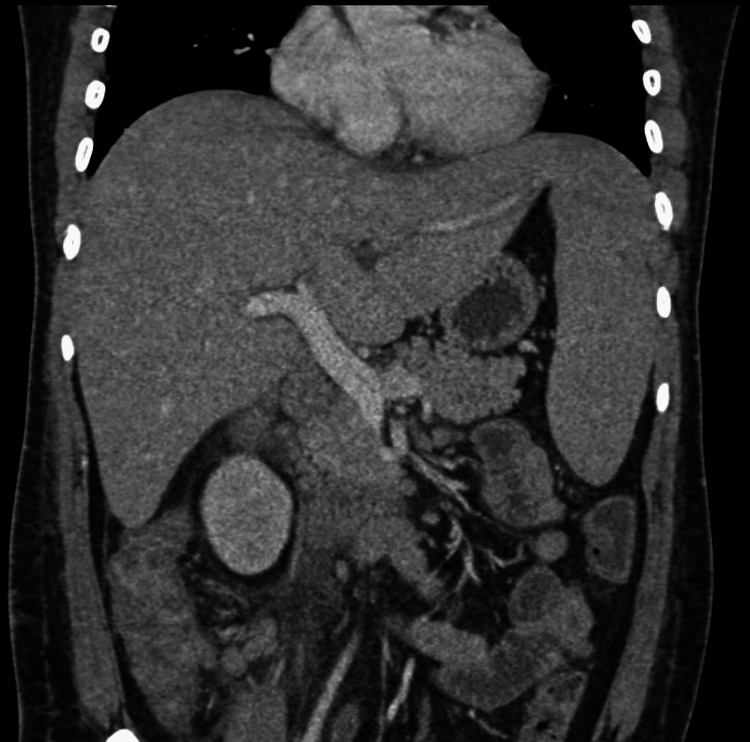
A computed tomography scan of the abdomen (coronal section) showing hepatosplenomegaly The liver was measured to be 20 cm (craniocaudal length in the midclavicular line). The spleen was measured to be 15 cm (craniocaudal length).

**Figure 2:**
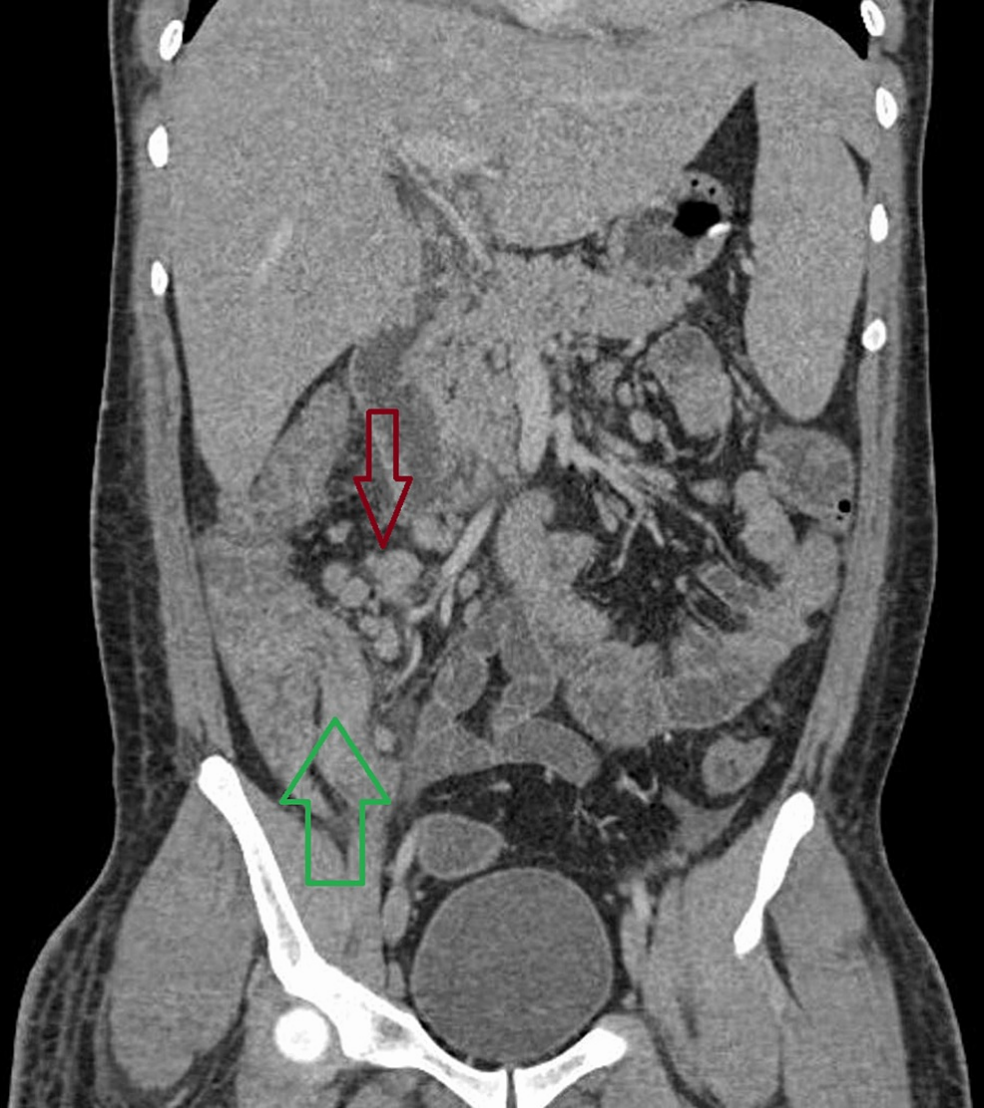
A computed tomography scan of the abdomen (coronal section) shows ileocecal thickening (green arrow) and multiple enlarged mesenteric lymph nodes in the right iliac fossa (red arrow) Circumferential wall thickening involving the terminal ileum, ileocecal junction, cecum, proximal appendix, and ileocecal valve causing narrowing of the ileocecal junction (green arrow) (maximum thickness being 6.5 mm). Extensive surrounding fat stranding is seen in the right iliac fossa. Multiple enlarged mesenteric lymph nodes are also seen (red arrow), the largest being 2.1 X 1.7 cm.

By the night, his O_2_ requirement had increased to 8 L/min by face mask. A CT of the thorax and abdomen done urgently revealed patches of ground glass opacities in bilateral lung fields with few non-necrotic mediastinal nodes, hepatosplenomegaly (Figure 1), thickened ileocecal junction with multiple enlarged non-necrotic and a few necrotic mesenteric lymph nodes in the right iliac fossa (Figure 2), peripancreatic region, mild ascites, and bilateral pleural effusion.

Findings of new-onset lung infiltrates with hypoxemia were suggestive of acute respiratory distress syndrome. Ileocecal thickening with mesenteric non-necrotic nodes and hepatosplenomegaly reinforced the diagnosis of enteric fever (with intestinal tuberculosis yet to be ruled out). Bone marrow aspiration showed hemophagocytes in moderately cellular marrow with normal maturation of cell lines.

Given persistent fever spikes (>101°F on days four and five), elevated liver enzymes, hyperferritinemia, hypertriglyceridemia, hypofibrinogenemia, a worsening clinical condition, and hemophagocytosis demonstrated on bone marrow aspirate, he was also started on dexamethasone (16 mg IV OD for HLH) on day five itself. On the same day, a blood culture was reported to have isolated *Salmonella typhi*, sensitive to gentamycin, amikacin, and meropenem; intermediately sensitive to azithromycin; and resistant to ceftriaxone, piperacillin, and tazobactam. No organism was isolated in urine or sputum cultures. Stool culture grew commensals.

Thus, we arrived at the diagnosis of HLH with acute respiratory distress syndrome secondary to typhoid fever. Sepsis with multiorgan dysfunction syndrome due to *Salmonella typhi* remained a competing diagnosis. But, worsening even on broad-spectrum antibiotics, a high H-score (203 points, i.e., 88%-93% probability of HLH) satisfied the Modified 2009 HLH criteria, heavily favouring HLH (Table 3).

**Table 4:** Patient parameters compared to Modified 2009 HLH criteria NK cell: natural killer cell

Modified 2009 HLH criteria	Patient parameters
At least three of the following	
Fever	Yes
Splenomegaly	Yes
Cytopenias in at least 2 cells lines	
Hemoglobin < 9 gm %	Yes (9.6 gm %)
Platelet <100000/cumm	Yes (29,000/cumm)
Absolute neutrophil count <1000/cumm	Yes (795/cumm)
Hepatitis	Yes
At least one of the following	
Ferritin elevation (>500 ng/mL)	Yes (>1500 ng/mL)
Elevated soluble CD25	Not available
Hemophagcytosis	Yes
Low/absent NK cell activity	Not available
Other supportive features (not required)	
Hypertriglyceridemia (Fasting >265mg/dL)	Yes (304 mg/dL)
Hypofibrinogenemia (<1500 mg/dL)	Yes (280 mg/dL)
Hyponatremia	No

The patient was continued on meropenem and dexamethasone. After receiving two days of dexamethasone, remarkable clinical improvement was noted. He became afebrile, and the O2 requirement decreased. The patient was on room air after the third day of dexamethasone. Cytopenias and liver enzymes started returning to normal. The patient received meropenem injections for 14 days and was discharged on oral dexamethasone tapered over six weeks. After six weeks, he was asymptomatic with normal hemograms and liver and kidney function tests. Stool culture done after six weeks grew commensals. Ultrasonography of the abdomen showed the disappearance of mesenteric lymphadenopathy and ileocecal thickening (thus ruling out co-existing intestinal tuberculosis).

## Discussion

Hemophagocytic lymphohistiocytosis (HLH) is a clinical syndrome characterised by an uncontrolled hyper-inflammatory state. It can be triggered by infections, autoimmune diseases, malignancies (called secondary HLH), or genetic defects in the immune system (called primary or familial HLH) [10]. The pathophysiology of both types involves hypercytokinemia (principally interleukin 2, interleukin 6, interferon-gamma, and tumour necrosis factor-alpha) and histiocyte activation [10,11]. Clinical (fever, hepatosplenomegaly) and laboratory manifestations (cytopenias, hemophagocytosis, hepatitis, hyperferritinemia, hypofibrinogenemia, and hypertriglyceridemia) of HLH are due to these high cytokine levels and activated histiocytes (macrophages) infiltrating the tissues [11].

Enteric fever includes typhoid fever (caused by *Salmonella typhi*) and paratyphoid fever (caused by *S. paratyphi A, B, and C*). The diagnosis should be considered in any patient with prolonged fever, abdominal symptoms (pain, diarrhoea, or constipation), a coated tongue, and hepatosplenomegaly, particularly in endemic areas. Non-specific symptoms like malaise, arthralgia, anorexia, headache, and rash may occur [12]. Severe or complicated disease usually manifests in the second and third weeks when left untreated or inappropriately treated, with continuing fever, anaemia, weight loss, gastrointestinal bleeding, encephalopathy, hepatitis, and nephritis [13].

In the case presented, clinical features of fever, abdominal symptoms, relative bradycardia, and hepatosplenomegaly raised the suspicion of enteric fever. Findings on CT imaging of the abdomen further supported this diagnosis, and a blood culture growing *Salmonella typhi* confirmed the diagnosis.

However, unlike the usual insidious deterioration of patients with untreated enteric fever, our patient started deteriorating rapidly despite broad-spectrum antibiotics. He was having high-spiking fevers, worsening cytopenias, hepatitis, and ARDS. Thus, HLH was suspected. Further investigations revealed hyperferritinemia, hypofibrinogenemia, and hypertriglyceridemia, and bone marrow aspirates showed hemophagocytes. A high H-score (203 points, i.e., 88%-93% probability of HLH) satisfied the Modified 2009 HLH criteria (Table 3), heavily favouring the diagnosis of HLH.

Since the patient's clinical condition was worsening despite receiving a culture- and sensitivity-appropriate antibiotic (meropenem), it was decided to add a corticosteroid (dexamethasone 10mg /m2 IV OD) as HLH-directed immunosuppression. The patient showed remarkable recovery. Antibiotics were continued for the duration of 14 days, and dexamethasone was slowly tapered off. In our patient, early diagnosis of HLH complicating typhoid fever was important for starting appropriate treatment and recovery.

While our patient required HLH-directed immunosuppression in addition to antibiotics, it should be noted that cases of infection-associated HLH may resolve by treating the infectious trigger alone. Whether to add immunosuppression and, if yes, how much immunosuppression depends on the clinical condition and organ dysfunction. There is no "one size fits all" treatment [6,7]. Non et al. reported a case of a 21-year-old female with typhoid fever complicated by HLH and rhabdomyolysis who improved with antibiotics and supportive care (without immunosuppression) [14]. Sánchez-Moreno et al. did a literature review for cases of typhoid/enteric fever complicated by HLH and found 11 such patients aged more than 12 years. Out of these 11 patients, only two needed immunosuppression in addition to antibiotics for recovery. One patient received dexamethasone, while the other received intravenous immune globulin (IVIG)+ dexamethasone as immunosuppression [8]. Ray et al. also described two patients with HLH secondary to typhoid fever. One of these recovered with antibiotics alone, while the other required steroids in addition to antibiotics [15]. George et al. (2018) also described a case of typhoid fever complicated by HLH that recovered with antibiotics alone [16].

There are only a few case reports of HLH secondary to typhoid or enteric fever in adults. Attributing signs of HLH (like leukopenia, anaemia, hepatitis, fever, and hepatosplenomegaly) to severe or complicated enteric fever may be one of the reasons for the late or missed diagnosis of HLH.

In addition to enteric fever, HLH has also been reported as a complication of other infections, like dengue fever [17].

## Conclusions

Hemophagocytic lymphohistiocytosis (HLH) is a rare but serious complication of enteric or typhoid fever. Physicians must possess a high index of suspicion for diagnosing HLH in patients with infections presenting with fever and cytopenias that are worsening even after appropriate antimicrobial and supportive treatment. Early diagnosis and treatment prevent catastrophic outcomes like multiorgan dysfunction and death. While treating patients with infection-associated HLH, the decision to add immunosuppression to infection-directed treatment has to be tailored according to clinical conditions and organ dysfunction.
